# Sex differences in quality indicator attainment for myocardial infarction: a nationwide cohort study

**DOI:** 10.1136/heartjnl-2018-313959

**Published:** 2018-11-23

**Authors:** Chris Wilkinson, Owen Bebb, Tatendashe B Dondo, Theresa Munyombwe, Barbara Casadei, Sarah Clarke, François Schiele, Adam Timmis, Marlous Hall, Chris P Gale

**Affiliations:** 1 Department of Clinical and Population Sciences, Leeds Institute of Cardiovascular and Metabolic Medicine, University of Leeds, Leeds, UK; 2 Division of Cardiovascular Medicine, British Heart Foundation Centre of Research Excellence, University of Oxford, Oxford, UK; 3 NIHR Oxford Biomedical Research Centre, Oxford University Hospitals NHS Foundation Trust, Oxford, UK; 4 Royal Papworth Hospital, Cambridge, UK; 5 Department of Cardiology, University Hospital Jean Minjoz, Besancon, France; 6 NIHR Cardiovascular Biomedical Research Unit, Barts Health Centre London, London, UK

**Keywords:** coronary artery disease, acute myocardial infarction, epidemiology, quality and outcomes of care, healthcare delivery

## Abstract

**Aim:**

To investigate sex differences in acute myocardial infarction (AMI) guideline-indicated care as defined by the European Society of Cardiology (ESC) Acute Cardiovascular Care Association (ACCA) quality indicators.

**Methods:**

Nationwide cohort study comprising 691 290 AMI hospitalisations in England and Wales (n=233 hospitals) from the Myocardial Ischaemia National Audit Project between 1 January 2003 and 30 June 2013.

**Results:**

There were 34.5% (n=238 489) women (median age 76.7 (IQR 66.3–84.0) years; 33.9% (n=80 884) ST-elevation myocardial infarction (STEMI)) and 65.5% (n=452 801) men (median age 67.1 (IQR 56.9–77.2) years; 42.5% (n=192 229) STEMI). Women less frequently received 13 of the 16 quality indicators compared with men, including timely reperfusion therapy for STEMI (76.8% vs 78.9%; p<0.001), timely coronary angiography for non-STEMI (24.2% vs 36.7%; p<0.001), dual antiplatelet therapy (75.4% vs 78.7%) and secondary prevention therapies (87.2% vs 89.6% for statins, 82.5% vs 85.6% for ACE inhibitor/angiotensin receptor blockers and 62.6% vs 67.6% for beta-blockers; all p<0.001). Median 30-day Global Registry of Acute Coronary Events risk score adjusted mortality was higher for women than men (median: 5.2% (IQR 1.8%–13.1%) vs 2.3% (IQR 0.8%–7.1%), p<0.001). An estimated 8243 (95% CI 8111 to 8375) deaths among women could have been prevented over the study period if their quality indicator attainment had been equal to that attained by men.

**Conclusion:**

According to the ESC ACCA AMI quality indicators, women in England and Wales less frequently received guideline-indicated care and had significantly higher mortality than men. Greater attention to the delivery of recommended AMI treatments for women has the potential to reduce the sex-AMI mortality gap.

**Video 1:** 

## Introduction

Women now account for more new cases of cardiovascular disease than men; in 2015 the incidence of cardiovascular disease among women was 5.3 million across 47 European Society of Cardiology (ESC) member countries.^1^ For acute myocardial infarction (AMI), women have higher early mortality rates and lower longer term survival than men.^2 3^ Women with AMI are more likely to have non-chest pain symptoms,^4^ longer delays in seeking medical care following symptom onset,^5^ myocardial infarction with non-obstructive coronary arteries (MINOCA)^6^ and a different comorbidity burden.^7^ However, such factors may not fully explain their disadvantaged outcomes. Research using the Swedish Web-system for Enhancement and Development of Evidence-based care in Heart disease Evaluated According to Recommended Therapies (SWEDEHEART) registry found that the delivery of cardiovascular care, but not patient demographics or medical history accounted for sex differences in excess mortality following AMI.^8^

The ESC Acute Cardiovascular Care Association (ACCA) recently developed and externally validated a suite of quality indicators (QI) for AMI.^9–11^ These dovetail many of the ESC guidelines class I level A recommendations for the management of ST-elevation myocardial infarction (STEMI) and non-STEMI (NSTEMI), and are inversely associated with 30-day and 3-year mortality.^10 11^ Given the knowledge gap with regard to the precise nature and full extent of the impact of sex-dependent inequalities in AMI care, we mapped the ESC ACCA QIs to men and women hospitalised in England and Wales with AMI. We aimed to identify where in the pathway of AMI care greater attention may be required to deliver ESC guideline-recommended interventions and therefore improve cardiovascular outcomes.

## Methods

### Setting and design

The analyses used data from the Myocardial Ischaemia National Audit Project (MINAP), a comprehensive registry of acute coronary syndrome hospitalisations in England and Wales mandated by the Department of Health.^12^ Each MINAP entry provides patient demographic and clinical details of the patient journey across 122 data items. Data collection and management have been described previously.^12^

**Figure 1:**
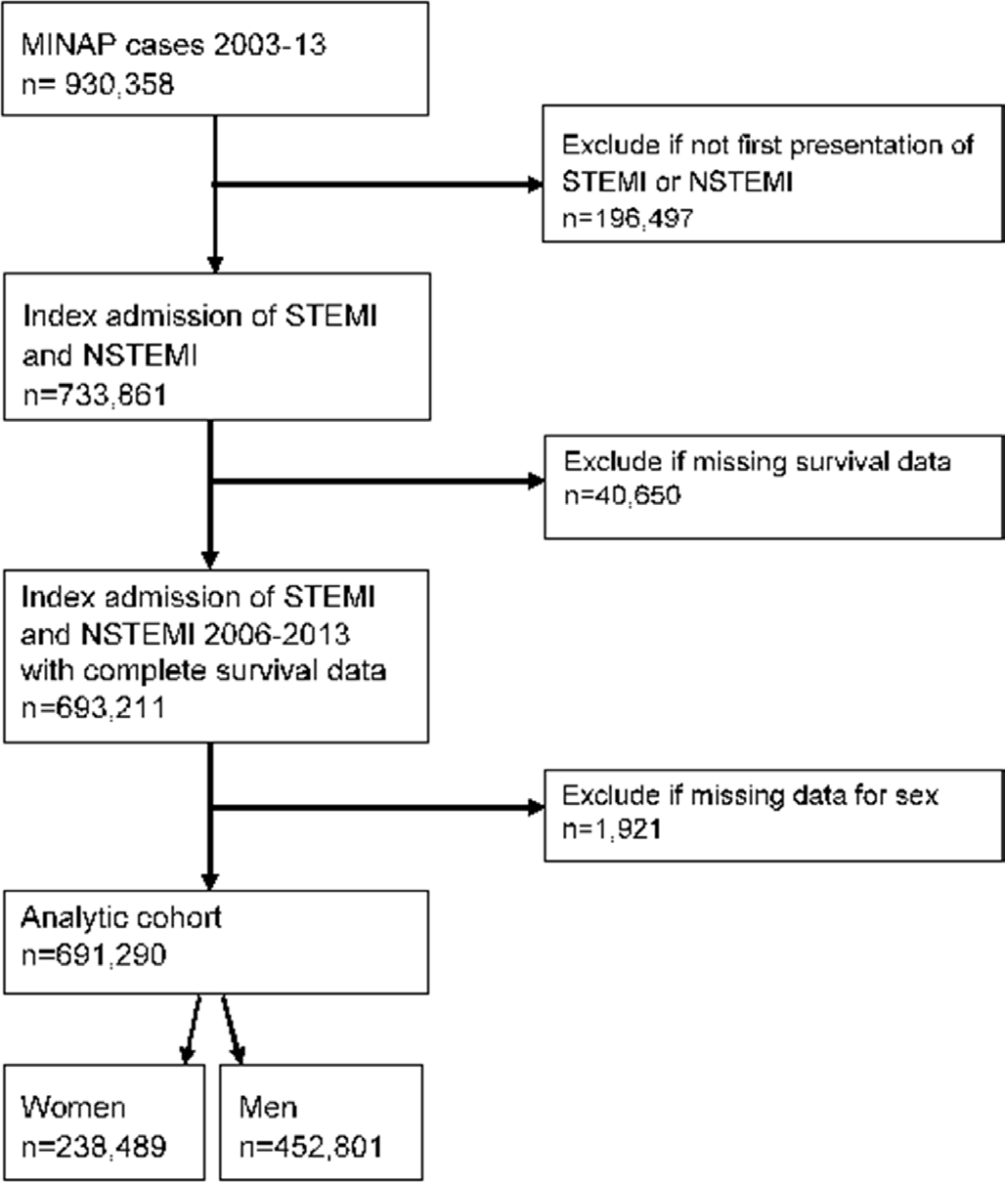
Strengthening the Reporting of Observational Studies in Epidemiology (STROBE) diagram of the analytical cohort. MINAP, Myocardial Ischaemia National Audit Project; NSTEMI, non-STEMI; STEMI, ST-elevation myocardial infarction.

Patients aged 18 years or over who were admitted to one of 233 hospitals between 1 January 2003 and 30 June 2013 with a discharge diagnosis of AMI (STEMI and NSTEMI) were included in the study (n=693 211). In the case of multiple admissions, the earliest record was used to reduce potential bias caused by treatments associated with prior admissions of AMI. Those with missing sex were excluded, leaving an analytical cohort of 691 290 (figure 1). Variables included demographics (age, sex, year of admission), cardiovascular risk factors (history of ischaemic heart disease (IHD), hypertension, diabetes mellitus, dyslipidaemia, smoking, peripheral vascular disease (PVD), chronic kidney disease (CKD), cerebrovascular disease family history of IHD), other medical history (heart failure (HF), asthma or chronic obstructive pulmonary disease (COPD)) and clinical characteristics (heart rate, systolic blood pressure (BP), out-of-hospital cardiac arrest, creatinine, ST-segment deviation, Killip class) at the time of hospitalisation. Vital status and date of all-cause mortality were linked from the Office for National Statistics, and provided as part of the anonymised patient-level data set.

Ethical approval for this study was not required under National Health Service (NHS) research governance arrangements for the secondary use of anonymised patient data. The National Institute for Cardiovascular Outcomes Research, including the MINAP database, has support under section 251 of the NHS Act 2006 to use patient information for medical research without consent (NIGB: ECC1-06(d)/2011). The study was conducted in compliance with the Declaration of Helsinki.

### Quality indicators

The ESC ACCA has set out 20 QIs for measuring attainment of guideline-indicated AMI care.^9^ These indicators cover seven domains, including the evaluation of: (1) centre organisation, (2) reperfusion / invasive strategy, (3) in-hospital risk assessment, (4) antithrombotic treatment during hospitalisation, (5) secondary prevention discharge treatments, (6) patient satisfaction, and (7) composite QIs, and Global Registry of Acute Coronary Events (GRACE) risk score adjusted 30-day mortality.

Numerators and denominators corresponding to the components of the QIs were taken from relevant MINAP data fields. An individual would be deemed ineligible if a QI was listed within MINAP as contraindicated, not indicated, not applicable, declined by the patient or was deemed inappropriate. If a patient was eligible for treatment, but had missing receipt of treatment data, a default imputation strategy was adopted where they would be treated as not having received the treatment.

Attainment of four QIs could not be calculated from the MINAP data fields: discharge prescription of high-intensity statins, documentation of GRACE or Can Rapid Risk Stratification of Unstable Angina Patients Suppress Adverse Outcomes with Early Implementation of the ACC/AHA Guidelines score in the medical notes and patient satisfaction. QIs that are part of routine UK processes were scored at 100% attainment, including assessment of time to reperfusion, participation in a regular registry, prehospital activation of the catheter laboratory and a single emergency phone number for medical triage.^11^ For domain 6, patient-reported experience is not recorded. We therefore report provision of dietary advice and cardiac rehabilitation referral.

Domain 7 specifies the use of composite scores including an opportunity-based and all-or-none score. The opportunity-based score was derived by dividing the total number of interventions received, by the number that the patient was eligible for. The nine measures available within MINAP that were included were: the centre is part of a network organisation; proportion of patients receiving reperfusion therapy among those eligible (STEMI, and first medical contact within 12 hours of onset of pain); coronary angiography in high-ischaemic-risk STEMI and NSTEMI without contraindications; assessment of left ventricular (LV) ejection fraction before hospital discharge; low-dose aspirin (unless high bleeding risk or oral anticoagulation); P2Y_12_ inhibition; ACE inhibitor (ACEi) or angiotensin receptor blocker (ARB) in patients with clinical evidence of HF or evidence of moderate/severe LV systolic dysfunction (LVSD) on echocardiography; beta-blockers (unless contraindicated) in patients with clinical evidence of HF or moderate/severe LVSD; and  prescription of a statin.

The all-or-none composite score was derived by categorising care receipt into optimal (all interventions received) or suboptimal (missed one or more care interventions for which the patient was eligible). For patients without HF, the measures included were receipt of low-dose aspirin, P2Y_12_ inhibitor and statin. For those with HF (defined as an ejection fraction of <40% or presence of HF symptoms) receipt of beta-blockers and ACEi/ARB were included in addition, making five variables in total.

### Statistical analyses

Baseline characteristics were described using numbers and percentages, medians and IQR or means with SDs. Comparisons were made using Χ^2^, t-test or Mann-Whitney U test. The proportion (%) of patients in whom each QI was attained of those who were eligible is reported.

Given MINAP does not directly record a patient’s baseline clinical risk, we calculated the mini-GRACE risk score, which has been validated for use within the registry.^13^ This comprises age, cardiac arrest, ST-segment deviation, elevated cardiac enzymes, systolic BP, heart rate, loop diuretic (as a surrogate for Killip class II–IV) and creatinine (as a surrogate for CKD).^13 14^ Each patient’s ischaemic risk was categorised in line with ESC guidance.^15^

To estimate 30-day GRACE adjusted mortality, we used the predicted probabilities derived from a logistic regression model where the dependent variable was 30-day mortality and the independent variable was each patient’s GRACE risk score. To estimate the number of potentially preventable deaths had QI attainment been equal between men and women, we calculated the GRACE risk adjusted OR for the association of the opportunity-based composite score with 30-day mortality using a logistic regression model. This was based on complete cases analysis. The OR corresponding to a single unit change in the composite opportunity-based score was converted to correspond to a change equivalent to the difference between median attainment levels of the score between women and men (by raising the OR to the power of the difference). The percentage change in mortality was then multiplied by the observed number of deaths in women to estimate the number of deaths that may have been prevented, had QI attainment been equal. Sensitivity analyses were performed using imputed data and fully adjusted models (online supplementary table 1).

Inverse probability weighting propensity score analysis was used to account for systematic differences between men and women (ie, differences in baseline characteristics). A series of Royston-Parmar flexible parametric survival models were then fitted to determine the extent to which year of admission, Index of Multiple Deprivation (IMD) score, GRACE risk score, comorbidities, risk factors and QIs explained the difference in 30-day survival between men and women (online supplementary section 1). The analyses were undertaken overall for the AMI group, as well as stratified by AMI subtype. Stata MP V.14.0 (StataCorp, USA) was used for analysis, with statistical significance determined at 5%.

10.1136/heartjnl-2018-313959.supp1 Supplementary file 1

## Results

### Patient characteristics

The analytical cohort comprised 238 489 women (34.5%) and 452 801 men (65.5%). Women were, on average, 9.6 years older and more frequently had NSTEMI than men (66.1% vs 57.5%, p<0.001) (table 1). Fewer women were smokers or ex-smokers than men, and there was a lower proportion with previous coronary revascularisation among women than men. Women had a greater prevalence of angina, hypertension, cerebrovascular disease, COPD or asthma, renal failure and HF, and a lower prevalence of previous AMI, hypercholesterolaemia and PVD compared with men (all p<0.001).

**Table 1:** Baseline characteristics of patients hospitalised with acute myocardial infarction, according to sex

Variable		Total cohort n**=**691 290	Women n=238 489 (34.5%)	Men n=452 801 (65.5%)	P values	Missing n (%)
Age (years)	Median (IQR)	70.7 (59.4–80.1)	76.7 (66.3–84.0)	67.1 (56.9–77.2)	<0.001	1025 (0.1)
Discharge diagnoses						
STEMI	n (%)	273 113 (39.5)	80 884 (33.9)	192 229 (42.5)	<0.001	0 (0)
NSTEMI	n (%)	418 177 (60.5)	157 605 (66.1)	260 572 (57.5)	<0.001	0 (0)
Medical history						
Diabetes mellitus	n (%)	122 185 (19.4)	44 044 (20.3)	77 877 (18.9)	<0.001	62 755 (9.1)
Current/ex-smoker	n (%)	390 891 (62.2)	104 570 (49.2)	285 349 (68.9)	<0.001	14 915 (2.2)
Ischaemic heart disease	n (%)	228 928 (36.4)	78 390 (36.2)	150 057 (36.5)	0.018	66 896 (9.7)
Previous myocardial infarction	n (%)	136 445 (21.9)	42 833 (20.0)	93 310 (22.9)	<0.001	57 597 (8.3)
Previous angina	n (%)	169 404 (27.5)	60 612 (28.6)	108 468 (27.0)	<0.001	64 084 (9.2)
Previous PCI	n (%)	46 586 (9.0)	11 444 (5.5)	35 056 (8.8)	<0.001	71 596 (10.3)
Previous CABG surgery	n (%)	35 118 (5.8)	7252 (3.5)	27 819 (7.0)	<0.001	70 439 (10.2)
Hypertension	n (%)	302 309 (48.7)	117 383 (54.8)	184 291 (45.5)	<0.001	58 415 (8.4)
Peripheral vascular disease	n (%)	27 615 (4.6)	8806 (4.2)	18 956 (4.9)	<0.001	73 948 (10.7)
Cerebrovascular disease	n (%)	51 853 (8.6)	21 263 (10.2)	30 492 (7.7)	<0.001	73 954 (10.7)
COPD or asthma	n (%)	89 192 (14.9)	36 695 (17.7)	52 341 (13.4)	<0.001	72 369 (10.4)
Chronic renal failure	n (%)	31 015 (5.1)	11 503 (5.5)	19 446 (4.9)	<0.001	72 435 (10.4)
Chronic heart failure	n (%)	33 799 (5.6)	15 059 (7.2)	18 694 (4.7)	<0.001	71 904 (10.4)
Hypercholesterolaemia	n (%)	193 576 (32.3)	63 372 (30.9)	129 495 (33.0)	<0.001	63 794 (9.2)
Admission characteristics						
Heart rate (beats/min)	Mean (SD)	81.9 (23.2)	84.9 (23.7)	80.3 (22.7)	<0.001	131 679 (19.0)
Systolic blood pressure (mm Hg)	Mean (SD)	139.2 (29.0)	140.2 (30.6)	138.7 (28.1)	<0.001	133 675 (19.3)
Prehospital cardiac arrest	n (%)	13 244 (2.0)	3290 (1.5)	9925 (2.3)	<0.001	44 865 (6.5)
Creatinine (μmol/L)	Mean (SD)	104.0 (61.1)	96.5 (57.7)	107.9 (62.5)	<0.001	308 817 (44.5)
GRACE risk score category						
Low (<109)	n (%)	134 380 (44.1)	31 869 (30.2)	102 340 (51.4)	<0.001	388 162 (56.0)
Intermediate (109–140)	n (%)	90 224 (29.6)	35 078 (33.2)	55 069 (27.6)		
High (>140)	n (%)	80 445 (26.4)	38 570 (36.6)	41 826 (21.0)		
Preadmission medication						
Aspirin	n (%)	153 311 (24.5)	54 759 (25.8)	98 256 (24.0)	<0.001	25 995
Beta-blocker	n (%)	136 230 (28.6)	48 518 (29.7)	87 511 (28.1)	<0.001	175 309
Statin	n (%)	204 489 (41.6)	69 054 (40.9)	135 116 (41.9)	<0.001	159 559
ACEi or ARB	n (%)	170 523 (35.9)	61 353 (37.6)	108 909 (35.1)	<0.001	176 395
Thienopyridine	n (%)	39 331 (14.3)	13 062 (13.9)	26 224 (14.5)	<0.001	398 166

Comparisons were made using Χ^2^ for categorical data, t-test for normally distributed continuous data and Mann-Whitney U test for non-normally distributed categorical data.

ACEi, ACE inhibitor; ARB, angiotensin II receptor blocker; CABG, coronary artery bypass graft; COPD, chronic obstructive pulmonary disease; GRACE, Global Registry of Acute Coronary Events; NSTEMI, non-STEMI; PCI, percutaneous coronary intervention; STEMI, ST-elevation myocardial infarction.

Women less frequently had a prehospital cardiac arrest than men (1.5% vs 2.3%, p<0.001), and more women were classified as high ischaemic risk according to the GRACE score (36.6% vs 21.0%, p<0.001). Prior to hospitalisation, women were more likely to be taking aspirin, beta-blockers and ACEi/ARBs, and less likely to be taking statins or P2Y_12_ inhibitors compared with men (all p<0.001).

### Quality indicators

**Figure 2:**
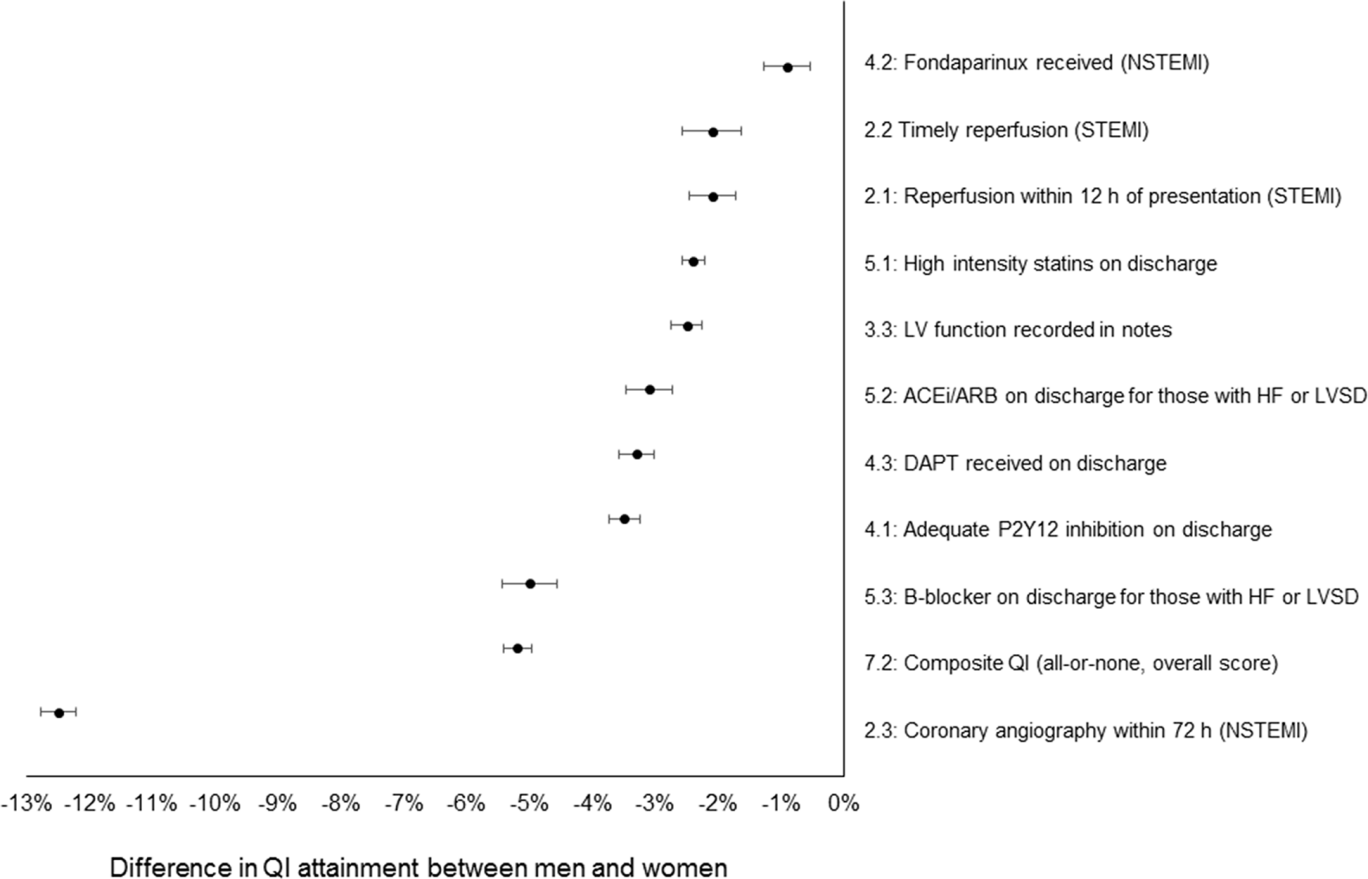
Mean difference in QI attainment between men and women. ACEi, ACE inhibitor; ARB, angiotensin receptor blocker; DAPT, dual antiplatelet therapy; HF, heart failure; LV, left ventricular; LVSD, left ventricular systolic dysfunction; NSTEMI, non-STEMI; QI, quality indicator; STEMI, ST-elevation myocardial infarction.

Data were available for six of the seven domains of care, covering 16 of the 20 QIs. For three QIs, a score of 100% was assigned to all patients. Of the remaining 13 QIs, there was significantly lower attainment for women compared with men (table 2, figure 2).

**Table 2:** Attainment of the ESC ACCA quality indicators for acute myocardial infarction, according to sex

Quality indicator	Type of quality indicator	Patients eligible (n)	Total receiving care, n (%) n**=**691 290	Women receiving care, n (%) n=238 489	Men receiving care, n (%) n=452 801	P values
1. Centre organisation						
1.1. Centre organisation: part of network	Main					
1.1a. Single emergency phone number to allow medical triage		691 290		100	100	
1.1b. Prehospital interpretation of the ECG		365 927	247 940 (67.8)	80 942 (67.1)	166 998 (68.1)	<0.001
1.1c. Prehospital activation of the catheter lab		691 290		100	100	
1.2. Routine assessment of times to reperfusion	Secondary	691 290		100	100	
1.3. Participate in regular registry	Secondary	691 290		100	100	
2. Reperfusion/invasive strategy						
2.1. Reperfusion within 12 hours of presentation (STEMI)	Main	177 418	153 394 (86.5)	40 577 (84.9)	112 817 (87.0)	<0.001
2.2. Timely reperfusion (STEMI)	Main	157 352	123 282 (78.3)	32 007 (76.8)	91 275 (78.9)	<0.001
2.2a. Fibrinolysis received within 30 min (PPCI centres and STEMI only)		8243	3310 (40.2)	862 (37.5)	2448 (41.1)	0.002
2.2b. Primary PCI received within 60 min (PPCI centres and STEMI only)		101 426	76 095 (75.0)	19 349 (73.3)	56 746 (75.6)	<0.001
2.2c. Primary PCI received within 120 min (non-PPCI and STEMI only)		47 201	43 622 (92.4)	11 739 (91.2)	31 883 (92.9)	<0.001
2.2d. Door-in to door-out time within 30 min (non-PPCI centres and STEMI only)		2173	631 (29.0)	131 (26.3)	500 (29.9)	0.118
2.3. Coronary angiography received within 72 hours (NSTEMI only)	Main	419 048	134 290 (32.0)	38 044 (24.2)	96 246 (36.7)	<0.001
2.4. Time from diagnosis to wire passage (STEMI), min (median, IQR min)	Secondary	80 734	44 (30–68)	46 (31–71)	44 (30–67)	<0.001
3. In-hospital risk assessment						
3.1. GRACE risk score recorded in notes	Main	NA	NA	NA	NA	NA
3.2. CRUSADE risk score recorded in notes	Main	NA	NA	NA	NA	NA
3.3. Left ventricular function recorded in notes	Main	691 290	336 228 (48.6)	111 991 (47.0)	224 237 (49.5)	<0.001
4. Antithrombotics during hospital stay						
4.1. Adequate P2Y_12_ inhibition on hospital discharge	Main	410 325	343 117 (83.6)	113 457 (81.3)	229 660 (84.8)	<0.001
4.2. Fondaparinux received (NSTEMI only)	Main	220 635	53 951 (24.4)	19 856 (24.2)	34 059 (24.6)	0.028
4.3. Dual antiplatelet therapy received on hospital discharge	Secondary	387 565	300 767 (77.6)	98 519 (75.4)	202 248 (78.7)	<0.001
5. Secondary prevention						
5.1. High-intensity statins on hospital discharge	Main	575 551	511 350 (88.8)	167 788 (87.2)	343 562 (89.6)	<0.001
5.2. ACEi/ARB on discharge for those with heart failure or LVSD	Secondary	167 221	141 152 (84.4)	53 597 (82.5)	87 555 (85.6)	<0.001
5.3. Beta-blocker on hospital discharge for those with heart failure or LVSD	Secondary	196 280	128 886 (65.7)	48 038 (62.6)	80 848 (67.6)	<0.001
6. Patient satisfaction						
6.1. Not applicable	Main	NA	NA	NA	NA	NA
7. Composite QI						
7.1. Composite QI (opportunity based) reported as percentage, median (IQR)	Main	691 290	75.0 (50.0–85.7)	71.4 (50.0–83.3)	80.0 (57.1–100.0)	<0.001
7.2. Composite QI (all-or-none, overall score)	Secondary	691 290	503 636 (72.9)	165 733 (69.5)	337 903 (74.6)	<0.001
7.2a. Composite QI (all-or-none, three measures) (%) patients with no heart failure or left ventricular ejection fraction ≤0.40	Secondary	467 825	374 122 (80.0)	116 760 (78.3)	257 362 (80.8)	<0.001
7.2b. Composite QI (all-or-none, five measures) (%) patients with heart failure or left ventricular ejection fraction ≤0.40	Secondary	223 465	129 514 (57.8)	48 973 (54.8)	80 541 (60.1)	<0.001
7.3. Mortality rate adjusted for GRACE risk score (median, IQR)*		305 549	3.1% (1.0–9.2)	5.2% (1.8–13.1)	2.3% (0.8–7.1)	<0.001
7.3. Mortality rate adjusted for GRACE risk score (mean, SD)*		305 549	7.6% (11.0)	9.9% (12.2)	6.3% (10.1)	<0.001

Comparisons were made using Χ^2^ for categorical data, t-test for normally distributed and Mann-Whitney U test for non-normally distributed data.

*Modelling based on complete cases only.

ACCA, Acute Cardiovascular Care Association; ACEi/ARB, ACE inhibitor/angiotensin II receptor blocker; CRUSADE, Can Rapid Risk Stratification of Unstable Angina Patients Suppress Adverse Outcomes with Early Implementation of the ACC/AHA Guidelines; ESC, European Society of Cardiology; GRACE, Global Registry of Acute Coronary Events; LVSD, left ventricular systolic dysfunction; NA, not applicable; NSTEMI, non-STEMI; PCI, percutaneous coronary intervention; PPCI, primary PCI; QI, quality indicator; STEMI, ST-elevation myocardial infarction.

#### Domain 1: centre organisation

All centres are part of a network with protocols to guide safe and efficient care, so scores of 100% were assigned. An ECG was performed and interpreted prehospital in more men than women (68.1% vs 67.1%, p<0.001).

#### Domain 2: reperfusion: invasive strategy

For STEMI presenting within 12 hours of symptom onset, less women than men received reperfusion therapy (84.9% vs 87.0%, p<0.001), and less women received reperfusion within the predefined time limits (76.8% vs 78.9%, p<0.001). The time from diagnosis to wire passage was longer for women than men (median 46 vs 44 min, p<0.001). For NSTEMI, fewer women than men received coronary angiography within 72 hours of admission to hospital (24.2% vs 36.7%, p<0.001).

#### Domain 3: in-hospital risk assessment

Less women than men had an inpatient evaluation of their LV ejection fraction (47.0% vs 49.5%, p<0.001).

#### Domain 4: antithrombotic treatment during hospitalisation

Less women than men with NSTEMI received fondaparinux (24.2% vs 24.6%, p=0.028). At hospital discharge, less women than men were prescribed P2Y_12_ inhibitors or dual antiplatelet therapies (both p<0.001).

#### Domain 5: secondary prevention: discharge treatment

Despite high attainment, less women than men were discharged on secondary prevention therapies. Where it was indicated, women less frequently than men received a statin, ACEi/ARB or beta-blocker (all p<0.001).

#### Domain 6: patient satisfaction

Less women than men received or were referred for cardiac rehabilitation (70.4% vs 76.7%, p<0.001) or received dietary advice (28.8% vs 32.0%, p<0.001).

#### Domain 7: composite and outcome QI

**Figure 3:**
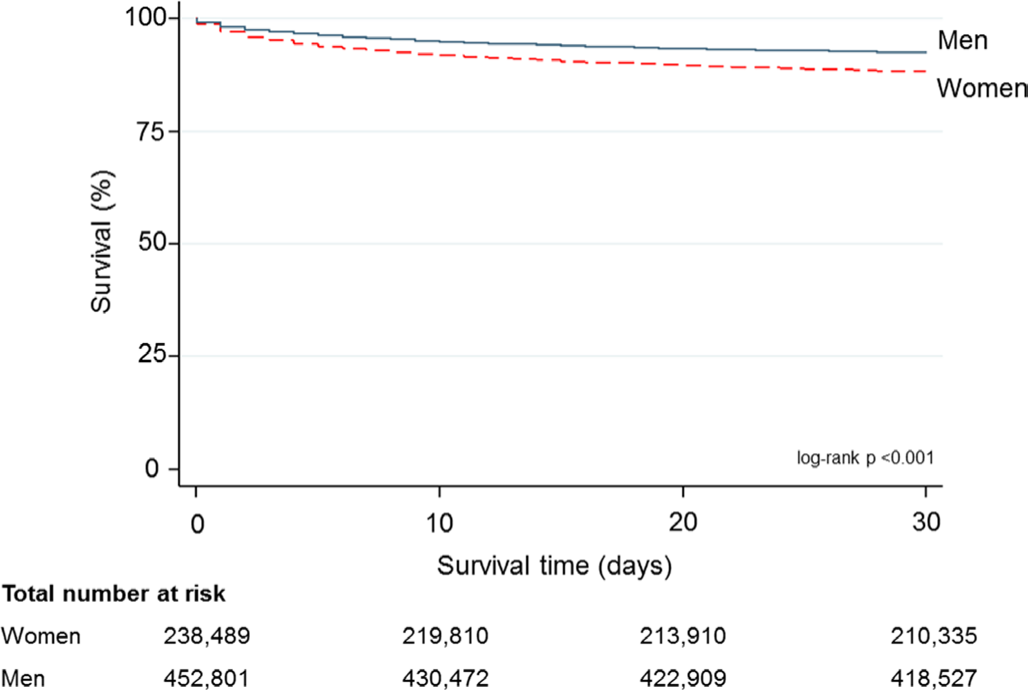
Kaplan-Meier survival curve of 30-day survival by sex.

Median attainment of the composite opportunity-based QI was higher for men than women (80.0% vs 71.4%, p<0.001). Similarly, the overall composite QI score was higher for men than women (74.6 vs 69.5, p<0.001). At 30 days, 28 004 women and 33 937 men had died (11.7% vs 7.5%, p<0.001). Thirty-day GRACE risk score adjusted mortality was higher among women than men (mean 9.9% vs 6.3%, p<0.001, median 5.2% vs 2.3%, p<0.001) (figure 3).

Overall, for a single percentage increase in the composite opportunity-based QI score, there was an associated 3.8% decline in the 30-day GRACE score adjusted mortality rate (OR=0.96, 95% CI 0.962 to 0.963; p<0.001). If women in the cohort (with complete GRACE score data) had the same composite opportunity-based score attainment as that of the men, an estimated 8243 (95% CI 8111 to 8375) deaths among women could potentially have been prevented at 30 days (online supplementary table 1).

### Propensity score analysis

**Figure 4:**
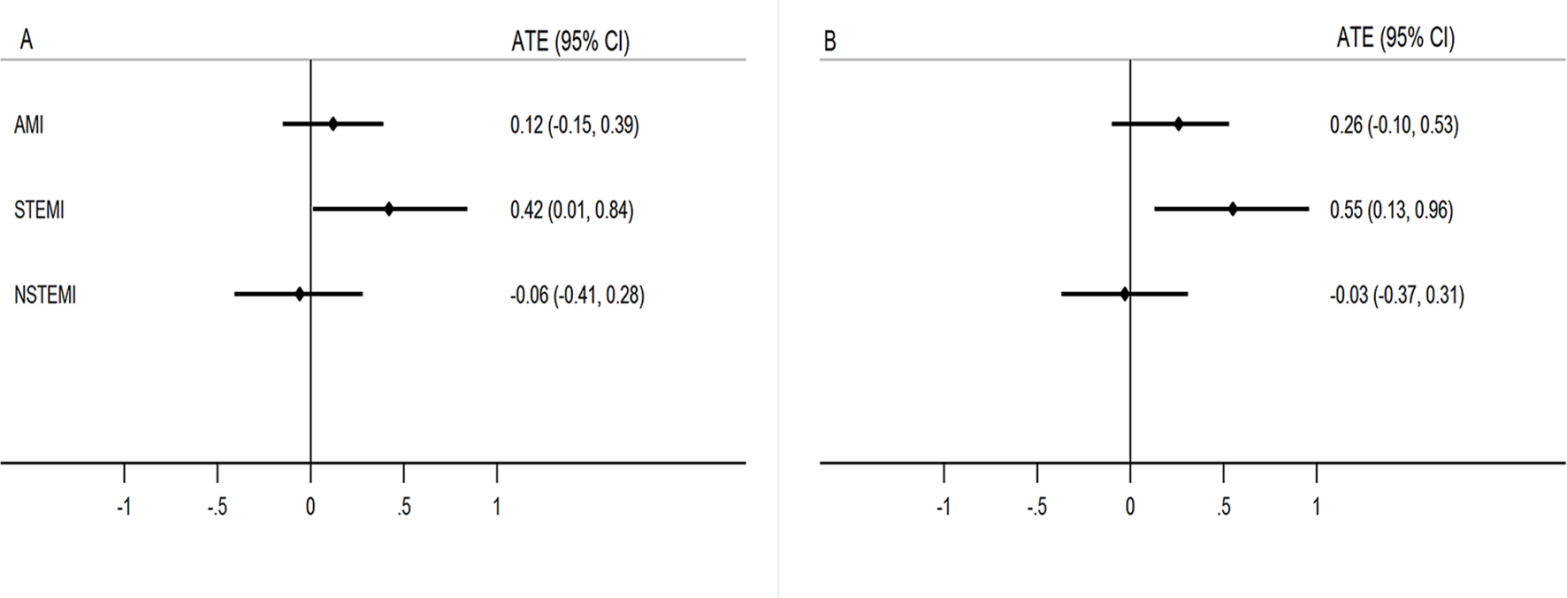
Propensity score analysis to show survival differences between men and women. (A) Average treatment effect (ATE) after weighting and adjustment for baseline characteristics (including Global Registry of Acute Coronary Events (GRACE) risk score). (B) ATE after further adjustment for the quality indicators. AMI, acute myocardial infarction; NSTEMI, non-STEMI; STEMI, ST-elevation myocardial infarction.

For STEMI, after weighting and adjustment for baseline characteristics (including GRACE score, supplementary tables 2 and 3), survival differences between men and women were evident (average treatment effect (ATE) 0.42, 95% CI 0.01 to 0.84; p=0.048) and remained after further adjustment for the QIs (ATE 0.55, 95% CI 0.13 to 0.96; p=0.010) (figure 4). For NSTEMI, no survival differences were observed after adjustment for baseline characteristics (ATE −0.06, 95% CI −0.41 to 0.28; p=0.717) or after further adjustment for QIs (ATE −0.03; 95% CI −0.37 to 0.31; p=0.850).

### Factors explaining the survival differences between the sexes

For STEMI, the QIs attenuated the difference in 30-day survival between men and women which was further reduced, but not eliminated by the addition of comorbidities, cardiovascular risk factors, year, demographics and GRACE score. For NSTEMI, the QIs also attenuated the difference in 30-day survival between men and women, and the difference was eliminated with the addition of comorbidities, cardiovascular risk factors and the GRACE score (online supplementary tables 4-6).

For inpatient mortality, clinical and demographic characteristics attenuated the difference between men and women, and the addition of the QIs eliminated the difference. In contrast, for those who survived to hospital discharge, the GRACE score attenuated the difference in survival to 30 days (online supplementary tables 7–9).

## Discussion

Using a nationwide clinical registry and an analytical cohort of nearly 700 000 patients with AMI, we found that the provision of care across the ESC ACCA QIs was lower for women than men. After adjustment for baseline ischaemic risk, women had higher rates of early mortality than men, and differences in the attainment of QIs mediated some of the differences in 30-day survival between men and women. While sex-dependent inequalities in cardiovascular care and outcomes have been reported previously, this study identifies where (and therefore how) deaths from AMI may be reduced among women.

Amounting evidence points towards the importance of health service factors in the clinical outcomes of women with AMI. Data from the SWEDEHEART registry showed that the lower survival among women than men was attenuated by adjustment for the use of pharmacotherapies and an invasive coronary strategy,^8^ which suggests that sex-dependent differences in AMI mortality are potentially modifiable through improved concordance with guideline-indicated care. Guideline recommendations are based on large randomised controlled trials which have demonstrated intervention efficacy^15 16^ and supported by real-world data verifying their effectiveness.^17 18^ Missed opportunities in the delivery of such care are associated with excess mortality, and potentially avoidable deaths.^17^ Attainment of the ACCA QIs is significantly associated with improved mortality,^11^ and their use in this study enabled us to identify inequities in provision of optimal care across the duration of clinical care.^9^

Whereas the combination of demographic and clinical characteristics, as well as the attainment of QIs, eliminated the sex-based difference in in-hospital mortality, the GRACE risk score had the greatest impact on sex-based differences in mortality at the landmark between discharge and 30 days. This suggests possible scope for improvement in in-hospital treatment for women with AMI. Provision of coronary angiography within 72 hours for NSTEMI showed the greatest sex discrepancy in QI attainment (12.5% lower for women than men). While the Early Invasive versus Selectively Invasive Management for Acute Coronary Syndromes (ICTUS) trial did not demonstrate a benefit for an early invasive strategy compared with a selective invasive strategy,^19^ in patients with high-risk NSTEMI early angiography has been shown to be associated with a lower risk of death and AMI,^20 21^ and therefore suboptimal attainment of this QI could potentially be associated with adverse outcomes. For STEMI, provision of timely reperfusion revealed a much smaller sex discrepancy which may, in part, be explained by differences in treatment pathways. During STEMI, the first encounter between patient and operator may be in the catheter laboratory, whereas in NSTEMI treatment decisions are made on the ward and may take greater account of comorbidities and frailty status. In STEMI, the small difference in delivery of primary percutaneous coronary intervention (PCI) between the sexes and the short duration of follow-up may explain the limited association between STEMI-specific QIs and differences in 30-day survival between men and women.^22^

Previous research has shown that women are less likely than men to receive guideline-indicated care,^8^ and that physicians tend to underestimate cardiovascular risk in women.^23^ Our findings imply that a combination of ‘biology and bias’ accounts for sex disparities in AMI treatment and outcome.^8 24 25^ Overall, the rates of delivery of interventions were high, and exceeded those elsewhere in Western Europe.^26^ There have been improvements in the use of AMI treatments, where provision of primary PCI for STEMI was 99.3% in 2016 in England.^27 28^ Yet, even in high functioning healthcare systems such as Sweden and the UK there appear to be systematic differences in the use of evidence-based medicine that disadvantages women.^8^ However, the QIs did not fully explain the sex differences in survival following AMI, and we also acknowledge sex differences in symptomatology, health-seeking behaviour^4 5^; comorbidity burden,^7^ vascular physiology, incidence of MINOCA^6^; and pharmacokinetics and pharmacodynamics.^29^

Whilst this study has strengths including the extent and quality of data, which allowed the interrogation of care according to the QIs, we recognise the limitations of our research. We followed the ACCA QI specification for the calculation of adjusted mortality,^9^ being mindful that other patient and hospital-specific influences were not accounted for in the modelling, and so residual confounding is likely. For the GRACE score we used surrogates for Killip class and creatinine.^13^ Similarly, ‘statin prescription’ was used as a surrogate for high-intensity statin, and any P2Y_12_ inhibitor for potent antiplatelet medication. MINAP does not record detail on coronary anatomy. Finally, we used the composite QIs to define optimal care and calculate the number of potentially avoidable deaths among women. However, this estimate may be imprecise due to limitations in data quality, and the fact that the QIs do not represent the full pathway of AMI care.

In conclusion, according to the ACCA QIs, women in England and Wales less frequently received guideline-indicated care and had significantly higher mortality rates than men. While the QIs did not fully explain sex differences in survival following AMI, greater attention to the delivery of recommended AMI treatments for women has the potential to reduce potentially avoidable deaths among women.

**Key messages**

**What is already known on this subject?**

- Women with acute myocardial infarction (AMI) have worse clinical outcomes than men. It is suggested that this relates to differences in comorbidity burden, clinical presentation and delivery of care.

**What might this study add?**

- We mapped the European Society of Cardiology AMI quality indicators to 691 290 patients using the UK Myocardial Ischaemia National Audit Project to investigate potential areas of suboptimal care provision for women, including timely reperfusion therapy for ST-elevation myocardial infarction (STEMI) and coronary angiography for non-STEMI, dual antiplatelet therapy and secondary prevention therapies. We found that attainment of each of these was lower, and 30-day Global Registry of Acute Coronary Events risk score adjusted mortality was higher in women. We estimate that 8243 deaths among women were potentially preventable had quality indicators attainment been equal between sexes.

**How might this impact on clinical practice?**

- Greater attention to guideline-indicated care has the potential to reduce the sex-dependent AMI mortality gap.
