# Supplementary material for: Sex differences in quality indicator attainment for myocardial infarction: a nationwide cohort study
Source: Heart. 2018 Nov 23;105(7):516–23. doi: 10.1136/heartjnl-2018-313959 (PMC6580739; doi:10.1136/heartjnl-2018-313959)
Supplement: Supplementary file 1 [file heartjnl-2018-313959supp001.docx]

**SUPPLEMENTARY MATERIAL**

**Sex differences in quality indicator attainment for acute myocardial infarction: a nationwide cohort study.**

Chris Wilkinson, Owen Bebb, Tatendashe B Dondo, Theresa Munyombwe, Barbara Casadei, Sarah Clarke, François Schiele, Adam Timmis, Marlous Hall, Chris P Gale

**Section 1: Survival-time inverse-probability weighting propensity score analysis**

The survival time inverse probability weighting propensity score method incorporated two models, a propensity score model for being male, which was used to derive inverse-probability weights using logistic regression analysis. This model included case mix, cardiovascular risk factors, QI and GRACE risk score variables. The second model was to determine the effect of sex on survival using the inverse-probability weights from model one to balance the covariate distribution between men and women. Two modelling approaches for the propensity scoring were undertaken. The first, a propensity score for being male was calculated using logistic regression analysis, including case mix, cardiovascular risk factor variables and GRACE risk score, only. The second propensity score was calculated using the same variables as for the first, plus the QIs. The survival models were then fitted using data weighted using weights from each of the propensity scoring approaches. Flexible parametric modelling was undertaken to explore the potential mediators between sex and 30-day survival. To do this, case mix variables were added to a univariable flexible parametric survival model which included sex. The statistical effect of patient demographics, GRACE risk score, co-morbidities and cardiovascular risk factors, and QIs on the survival differences between men and women was then determined by incrementally adding these variables to the model.

**Further detail**

The survival time inverse probability weighting propensity score analysis was undertaken to account for systematic differences between males and females, i.e. differences in baseline characteristics and receipt of care.^1^ A non-parsimonious multivariable logistic regression model was used for the treatment model and a Weibull survival model for the time to event model. To assess the effects of the difference in both baseline characteristics and treatment, two modelling approaches were adopted. In the first modelling approach,^2^ a propensity score for being male was calculated using a non-parsimonious multivariable logistic regression model, including patient demographics (deprivation (index of multiple deprivation score) and year of admission to hospital), cardiovascular risk factors (diabetes mellitus, hypercholesterolaemia, hypertension, smoking status, family history of coronary heart disease, chronic obstructive pulmonary disease, cerebrovascular disease, peripheral vascular disease, previous CABG, previous MI, previous angina), and adjusted mini-GRACE risk score (derived using variables: age, cardiac arrest, elevated enzyme, systolic blood pressure and heart rate at hospitalisation and creatinine) only. The survival model was then run on data weighted using the weights from the non-parsimonious multivariable logistic regression model and survival differences between males and females were determined. For the second modelling approach, a second non-parsimonious multivariable logistic regression model, including the same variables as for the first, plus the quality indicators (timely reperfusion (STEMI only), coronary angiography received within 72 hours (NSTEMI only), left ventricular function assessment, adequate P2Y_12_ inhibition on hospital discharge, fondaparinux received (NSTEMI only), dual antiplatelet therapy received on hospital discharge, high intensity statins on hospital discharge, ACEi/ARB and β blocker on hospital discharge for those with heart failure or left ventricular ejection fraction ≤0.40). A second survival model was then run on data weighted using the weights from the second propensity scoring model including quality indicators. In order to assess whether the weights constructed from the two non-parsimonious multivariable logistic regression models balanced the covariates between treated and control individuals standardised differences and variance ratios of the raw data versus weighted data were calculated (a perfectly balanced covariate has a standardised difference of zero and variance ratio of one). The weights adequately balanced the data and the balance checks are summarised in supplementary tables 1 and 2. Most of the standardised differences and variance ratios for variables in the weighted data were close to zero and one, respectively. The diagnostic assessments suggest that weighting by the inverse probability of treatment created a sample in which the prevalence of the case mix variables were similar between the male and female subjects. Treatment effects were estimated as average treatment effects (ATE) and average treatment effects on the treated (ATET). ATEs are defined as the population average of the contrast in outcomes when everyone gets the treatment and when no one gets the treatment. ATETs are defined as the mean difference in survival among the subjects that actually receive treatment. The analyses were carried out overall for AMI patients and stratified by the AMI phenotype.

1. Austin PC. The use of propensity score methods with survival or time‐to‐event outcomes: reporting measures of effect similar to those used in randomized experiments. Stat Med 2014;**33**(7):1242-1258.

2. Royston P, Lambert PC. *Flexible parametric survival analysis using Stata: beyond the Cox model*. StataCorp LP, College Station, Texas.: Stata Press; 2011.

**Table 1.** Sensitivity analyses for number of avoidable deaths associated among women associated with sub-optimal QI attainment.

| **Analytical cohort (n)** | **Model** | **Avoidable deaths, 95% CI** | **p value** | **%** |
| --- | --- | --- | --- | --- |
| Complete cases  (305,549) | Adjusted for GRACE | 8,243 (8,111-8,375) | <0.001 | 2.7% |
| Imputed data models (691,290) | Adjusted for GRACE | 20,062 (19,888-20,062) | <0.001 | 2.9% |
| Imputed data models (691,290) | Fully adjusted* | 19,540 (19,365-19,540) | <0.001 | 2.8% |

*Model adjusted for GRACE score, final diagnosis, previous percutaneous coronary intervention, previous coronary artery bypass graft, congestive cardiac failure, chronic renal failure, hypercholesterolemia, chronic obstructive pulmonary disease, left ventricular systolic dysfunction, peripheral vascular disease, hypertension, previous angina, previous myocardial infarction, smoking history, diabetes, cerebrovascular disease.

**Table 2.** Balance check parameters using standardized differences and variance ratios

|  | **Standardized differences** | | **Variance ratios** | |
| --- | --- | --- | --- | --- |
| **Data** | **Raw** | **Weighted** | **Raw** | **Weighted** |
| Deprivation (IMD) |  |  |  |  |
| Least deprived (1) | ref | ref | ref | ref |
| 2 | 0.01 | 0.002 | 1.001 | 1.00 |
| 3 | 0.002 | -0.002 | 1.00 | 1.00 |
| 4 | -0.01 | -0.001 | 0.98 | 1.00 |
| Most deprived (5) | -0.02 | -0.004 | 0.97 | 0.99 |
| Year of admission | 0.01 | -0.004 | 1.01 | 1.01 |
| **Cardiovascular history** |  |  |  |  |
| Cerebrovascular disease | -0.09 | 0.0002 | 1.04 | 1.00 |
| Peripheral vascular disease | 0.04 | -0.01 | 1.17 | 0.97 |
| **Cardiovascular risk factors** |  |  |  |  |
| Diabetes | -0.03 | -0.002 | 0.95 | 1.00 |
| Chronic cardiac failure | -0.11 | 0.002 | 0.66 | 1.01 |
| Chronic renal failure | -0.05 | -0.002 | 0.84 | 0.99 |
| Hypercholesterolaemia | 0.05 | -0.002 | 1.04 | 1.00 |
| Hypertension | -0.19 | 0.01 | 1.01 | 1.00 |
| Current or ex-smoker | 0.42 | -0.01 | 0.85 | 1.01 |
| Asthma or COPD | -0.13 | -0.01 | 0.79 | 0.99 |
| Previous CABG | 0.16 | -0.01 | 1.91 | 0.98 |
| Previous MI | 0.07 | -0.003 | 1.11 | 1.00 |
| Previous angina | -0.03 | -0.002 | 0.97 | 1.00 |
| Previous PCI | 0.14 | -0.01 | 1.51 | 0.97 |
| **GRACE risk score** |  |  |  |  |
| Low | ref | ref | ref | ref |
| Intermediate | 0.18 | -0.003 | 1.19 | 1.00 |
| High | -0.41 | 0.01 | 1.06 | 1.00 |

**Abbreviations:** IMD, Index of multiple deprivation; COPD, chronic obstructive pulmonary disease; CABG, coronary artery bypass graft; MI,myocardial infarction; PCI, percutaneous coronary intervention; ref, reference category; GRACE, Global Registry Acute Coronary Events: ref; reference category

**Table 3.** Balance check parameters using standardized differences and variance ratios

|  | **Standardized differences** | | **Variance ratios** | |
| --- | --- | --- | --- | --- |
| **Data** | **Raw** | **Weighted** | **Raw** | **Weighted** |
| Deprivation (IMD) |  |  |  |  |
| Least deprived (1) | ref | ref | ref | ref |
| 2 | 0.01 | 0.002 | 1.01 | 1.00 |
| 3 | 0.002 | -0.003 | 1.002 | 1.00 |
| 4 | -0.01 | -0.001 | 0.98 | 1.00 |
| Most deprived (5) | -0.02 | -0.01 | 0.97 | 0.99 |
| Year of admission | 0.01 | -0.001 | 1.01 | 1.01 |
| **Cardiovascular history** |  |  |  |  |
| Cerebrovascular disease | -0.09 | -0.0001 | 0.77 | 1.00 |
| Peripheral vascular disease | 0.04 | -0.01 | 1.18 | 0.97 |
| **Cardiovascular risk factors** |  |  |  |  |
| Diabetes | -0.03 | -0.003 | 0.95 | 0.99 |
| Chronic cardiac failure | -0.11 | 0.001 | 0.66 | 1.00 |
| Chronic renal failure | -0.05 | -0.003 | 0.84 | 0.99 |
| Hypercholesterolaemia | 0.05 | -0.00002 | 1.04 | 1.00 |
| Hypertension | -0.19 | 0.01 | 1.01 | 1.00 |
| Current or ex-smoker | 0.42 | -0.01 | 0.85 | 1.01 |
| Asthma or COPD | -0.13 | -0.01 | 0.79 | 0.99 |
| Previous CABG | 0.16 | -0.01 | 1.91 | 0.98 |
| Previous MI | 0.07 | -0.002 | 1.11 | 1.00 |
| Previous angina | 0.07 | -0.002 | 1.11 | 1.00 |
| Previous PCI | 0.13 | -0.01 | 1.51 | 0.97 |
| **Quality indicators** |  |  |  |  |
| Timely reperfusion (STEMI only) | 0.16 | 0.001 | 0.98 | 1.00 |
| Coronary angiography within 72 hours (NSTEMI only) | 0.10 | -0.01 | 1.18 | 0.99 |
| Received ECG and admitted by ambulance | 0.06 | -0.004 | 1.00 | 1.00 |
| Left ventricular function assessment | 0.06 | -0.004 | 0.98 | 1.00 |
| P2Y_12_ inhibition on hospital discharge | 0.10 | -0.001 | 0.90 | 1.00 |
| Fondaparinux received (NSTEMI only) | -0.05 | -0.002 | 0.92 | 1.00 |
| Dual antiplatelet therapy received on hospital discharge | 0.10 | -0.001 | 0.93 | 1.00 |
| High intensity statins on hospital discharge | 0.11 | -0.002 | 0.86 | 1.00 |
| ACEi/ARB on hospital discharge | -0.07 | 0.003 | 0.93 | 1.00 |
| β blocker on hospital discharge | -0.06 | 0.01 | 0.93 | 1.01 |
| **GRACE risk score** |  |  |  |  |
| Low | ref | ref | ref | ref |
| Intermediate | 0.18 | -0.001 | 1.19 | 1.00 |
| High | -0.41 | 0.01 | 1.06 | 1.00 |

**Abbreviations:** IMD, Index of multiple deprivation; COPD, chronic obstructive pulmonary disease; CABG, coronary artery bypass graft; MI; myocardial infarction; PCI, percutaneous coronary intervention; ref, reference category; STEMI; ST Elevation Myocardial Infarction,; NSTEMI, Non-ST Elevation Myocardial Infarction; GRACE, GRACE, Global Registry Acute Coronary Events: ref; reference category, ACE, angiotensin converting enzyme; ARB, angiotensin receptor blocker.

**Table 4.** Impact of treatment and patient baseline characteristics on the observed sex differences in survival (AMI group)

|  |  | **30 day survival** |  |
| --- | --- | --- | --- |
| **Model number** | **Variables included** | **Hazard ratio (95% CI)** | **p-value** |
| Model 1 | Sex | 0.62 (0.60 to 0.65) | <0.001 |
|  | Sex + |  |  |
| Model 2 | Year of admission and IMD score | 0.62 (0.60 to 0.65) | <0.001 |
| Model 3 | Year of admission and IMD score + GRACE risk score | 0.88 (0.85 to 0.91) | <0.001 |
| Model 4 | Year of admission and IMD score + GRACE risk score + Comorbidities and risk factors | 0.92 (0.89 to 0.96) | <0.001 |
| Model 5 | Year of admission and IMD score + GRACE risk score + Comorbidities and risk factors + QIs* | 1.00 (0.96 to 1.04) | 0.923 |
| **Alternative model building order** | | | |
|  | Sex + |  |  |
| Model 6 | QIs | 0.77 (0.74 to 0.80) | <0.001 |
| Model 7 | QIs + Comorbidities and risk factors | 0.81 (0.78 to 0.85) | <0.001 |
| Model 8 | QIs + Comorbidities and risk factors + Year of admission and IMD score | 0.81 (0.78 to 0.85) | <0.001 |
| Model 9 | QIs + Comorbidities and risk factors + Year of admission and IMD score + GRACE risk score | 1.00 (0.96 to 1.04) | 0.923 |

**Abbreviations:** GRACE, Global Registry Acute Coronary Events. IMD, Index of multiple deprivation. QI, Quality Indicator

**Table 5:** Impact of treatment and patient baseline characteristics on the observed sex differences in survival (STEMI phenotype)

|  |  | **30 day survival** |  |
| --- | --- | --- | --- |
| **Model number** | **Variables included** | **Hazard ratio (95% CI)** | **p-value** |
| Model 1 | Sex | 0.47 (0.45 to 0.50) | <0.001 |
|  | Sex + |  |  |
| Model 2 | Year of admission and IMD score | 0.47 (0.45 to 0.50) | <0.001 |
| Model 3 | Year of admission and IMD score + GRACE risk score | 0.71 (0.67 to 0.75) | <0.001 |
| Model 4 | Year of admission and IMD score + GRACE risk score + Comorbidities and risk factors | 0.76 (0.72 to 0.81) | <0.001 |
| Model 5 | Year of admission and IMD score + GRACE risk score + Comorbidities and risk factors + QIs* | 0.88 (0.82 to 0.95) | 0.001 |
| **Alternative model building order** | | | |
|  | Sex + |  |  |
| Model 6 | QIs | 0.60 (0.56 to 0.64) | <0.001 |
| Model 7 | QIs + Comorbidities and risk factors | 0.67 (0.62 to 0.72) | <0.001 |
| Model 8 | QIs + Comorbidities and risk factors + Year of admission and IMD score | 0.67 (0.62 to 0.72) | <0.001 |
| Model 9 | QIs + Comorbidities and risk factors + Year of admission and IMD score + GRACE risk score | 0.88 (0.82 to 0.95) | 0.001 |

**Abbreviations:** GRACE, Global Registry Acute Coronary Events. IMD, Index of multiple deprivation. QI, Quality Indicator

**Table 6:** Impact of treatment and patient baseline characteristics on the observed sex differences in survival (NSTEMI phenotype)

|  |  | **30 day survival** |  |
| --- | --- | --- | --- |
| **Model number** | **Variables included** | **Hazard ratio (95% CI)** | **p-value** |
| Model 1 | Sex | 0.71 (0.68 to 0.74) | <0.001 |
|  | Sex + |  |  |
| Model 2 | Year of admission and IMD score | 0.71 (0.68 to 0.74) | <0.001 |
| Model 3 | Year of admission and IMD score + GRACE risk score | 0.98 (0.94 to 1.02) | 0.330 |
| Model 4 | Year of admission and IMD score + GRACE risk score + Comorbidities and risk factors | 1.012 (0.97 to 1.06) | 0.504 |
| Model 5 | Year of admission and IMD score + GRACE risk score + Comorbidities and risk factors + QIs* | 1.06 (1.01 to 1.12) | 0.024 |
| **Alternative model building order** | | | |
|  | Sex + |  |  |
| Model 6 | QIs | 0.87 (0.83 to 0.91) | <0.001 |
| Model 7 | QIs + Comorbidities and risk factors | 0.89 (0.85 to 0.94) | <0.001 |
| Model 8 | QIs + Comorbidities and risk factors + Year of admission and IMD score | 0.89 (0.84 to 0.93) | <0.001 |
| Model 9 | QIs + Comorbidities and risk factors + Year of admission and IMD score + GRACE risk score | 1.06 (1.01 to 1.12) | 0.024 |

**Abbreviations:** GRACE, Global Registry Acute Coronary Events. IMD, Index of multiple deprivation. QI, Quality Indicator

**Table 7:** Table showing mortality as an inpatient and at 30-days, stratified by sex

|  | **In-hospital mortality** | | **30-days (excluding in-hospital deaths)** | |
| --- | --- | --- | --- | --- |
|  | % (n) | | % (n) | |
| Men | 6.0% | (27,055) | 2.1% | (8,956) |
| Women | 10.2% | (24,072) | 2.9% | (6,115) |
| Total | 7.5% | (51,127) | 2.4% | (15,071) |

**Table 8:** Impact of treatment and patient baseline characteristics on the observed sex differences in survival (in-hospital deaths only considered)

| **Model number** | **Variables included** | **Odds ratio (95% CI)** | **p-value** |
| --- | --- | --- | --- |
| Model 1 | Sex | 0.57 (0.55-0.59) | <0.001 |
|  | Sex + |  |  |
| Model 2 | Year of admission and IMD score | 0.57 (0.55-0.59) | <0.001 |
| Model 3 | Year of admission and IMD score + GRACE risk score | 0.93 (0.90-0.97) | 0.001 |
| Model 4 | Year of admission and IMD score + GRACE risk score + Comorbidities and risk factors ǂ | 0.98 (0.94-1.02) | 0.222 |
| Model 5 | Year of admission and IMD score + GRACE risk score + Comorbidities and risk factors ǂ + inpatient QI’s * | 1.04 (1.00-1.09) | 0.057 |
| ǂ Co-morbidities and risk factors included were: final diagnosis, previous percutaneous coronary intervention, previous coronary artery bypass graft, congestive cardiac failure, chronic renal failure, hypercholesterolemia, chronic obstructive pulmonary disease, left ventricular systolic dysfunction, peripheral vascular disease, hypertension, previous angina, previous myocardial infarction, smoking history, cerebrovascular disease, diabetes.  * Inpatient QIs included in the model were: received ECG and admitted by ambulance, for patients with STEMI: had timely reperfusion, for patients with NSTEMI: Received angiogram within 72hrs admission, received LVSF assessment, for patients with NSTEMI: Received fondaparinux. | | | |

**Table 9:** Impact of treatment and patient baseline characteristics on the observed sex differences in survival (hospital survivors only considered)

| **Model number** | **Variables included** | **Odds ratio (95% CI)** | **p-value** |
| --- | --- | --- | --- |
| Model 1 | Sex | 0.73 (0.69-0.77) | <0.001 |
|  | Sex + |  |  |
| Model 2 | Year of admission and IMD score | 0.73 (0.69-0.77) | <0.001 |
| Model 3 | Year of admission and IMD score + GRACE risk score | 1.05 (0.99-1.12) | 0.129 |
| Model 4 | Year of admission and IMD score + GRACE risk score + Comorbidities and risk factors * | 1.06 (1.00-1.13) | 0.054 |
| Model 5 | Year of admission and IMD score + GRACE risk score + Comorbidities and risk factors * + composite QI | 1.16 (1.09-1.24) | <0.001 |
| * Co-morbidities and risk factors included were: final diagnosis, previous percutaneous coronary intervention, previous coronary artery bypass graft, congestive cardiac failure, chronic renal failure, hypercholesterolemia, chronic obstructive pulmonary disease, left ventricular systolic dysfunction, peripheral vascular disease, hypertension, previous angina, previous myocardial infarction, smoking history, cerebrovascular disease, diabetes. | | | |
